# A validated LC–MS/MS multi-method for the determination of 110 mycotoxins and plant toxins in cow milk and application to samples from Germany

**DOI:** 10.1007/s00216-025-06024-6

**Published:** 2025-08-07

**Authors:** Ahmed H. El-Khatib, Arnold Bahlmann, Christoph Hutzler, Stefan Weigel

**Affiliations:** Reference Centre for Food and Feed Analysis, German Federal Institute for Risk Assessment (BfR), Max‑Dohrn‑Str. 8‑10, 10589 Berlin, Germany

**Keywords:** QuEChERS, Emerging mycotoxins, Beauvericin, Enniatins, Quinolizidine alkaloids, Lupanine

## Abstract

Milk is widely consumed across all ages and is essential for dairy production. Milk quality and safety are largely influenced by the animal’s diet, which may contain feedborne contaminants. This can lead to multi-toxin exposure, potentially transferring toxins to milk and posing health risks to consumers. Currently, only aflatoxin M1 (AFM1) is regulated by the European Union in milk (maximum level (ML) = 0.05 µg/kg) but the (co-)occurrence of other toxins should also be investigated. In this work, a QuEChERS-based LC–MS/MS multi-method for the simultaneous determination of 72 mycotoxins and 38 plant toxins in raw cow milk was developed and validated according to the latest EU regulations. The method showed excellent recoveries (87% of the analytes have average recoveries within 70–120%) and precision (97% of the analytes have within-laboratory reproducibility ≤20%). Limit of quantification (LOQ) for AFM1 (0.0035 µg/kg) was ≤½ ML. The method was applied to 20 milk samples (15 conventional and 5 organic samples) from retail stores and local farms. The analysis of these milk samples showed the co-occurrence of the emerging mycotoxins beauvericin and enniatin B as well as the quinolizidine alkaloids (QAs) lupanine and 13a-hydroxylupanine in the majority of samples. The pyrrolizidine alkaloid senkirkine was also detected in almost half of the samples. The results revealed generally trace levels of mycotoxins and plant toxins. However, QAs (especially lupanine) were detected at much higher concentration levels than other toxin groups (especially in organic milk samples). The findings showed the co-occurrence of multiple toxins in the range of 2–12 toxins in every sample.

**Graphical Abstract:**

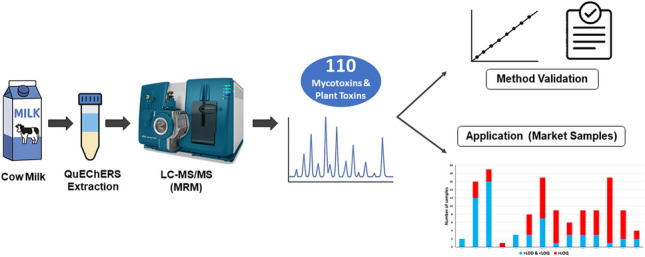

**Supplementary Information:**

The online version contains supplementary material available at 10.1007/s00216-025-06024-6.

## Introduction

Milk is one of the most consumed food products in all age groups and forms the basis of dairy products. The quality and safety of milk are significantly influenced by the animal’s diet, which can be composed of a complex mix of individual feed components (e.g., forages and concentrates). Each raw feed component can carry different contaminants, such as mycotoxins and plant toxins, leading to multi-toxin exposure in the overall animal diet [1–3]. This multitude of toxins and their metabolites and biotransformation products could finally find their way into the milk [4, 5]. Consequently, the presence of mycotoxins and plant toxins can pose health risks to consumers depending on the toxin, exposure level and duration, and the individual’s susceptibility. Several mycotoxins, such as aflatoxins, ochratoxin A, fumonisins, trichothecenes, and zearalenone, are well known for their potential genotoxicity, carcinogenicity, hepatotoxicity, nephrotoxicity, and/or immunotoxicity [6–8]. In addition, combined exposure to multiple mycotoxins may result in additive or synergistic toxic effects, posing an increased risk to human health [9]. Similarly, certain plant toxins such as pyrrolizidine alkaloids are hepatotoxic and genotoxic and have been linked to liver tumors [10, 11]. Newborns and infants are particularly vulnerable to exposure to these mycotoxins and plant toxins [10, 12].

Currently, only the mycotoxin aflatoxin M1 (AFM1) is regulated by the European Union in raw milk, heat-treated milk, and milk for the manufacture of milk-based products, with a maximum level (ML) of 0.05 µg/kg [13]. AFM1 is the less toxic hepatic transformation product of aflatoxin B1 (AFB1) and is the most frequently studied mycotoxin in milk [4, 14–16]. However, the presence of other aflatoxins (B1, B2, G1, G2, M2) in milk has also been reported [17–19]. Ochratoxin A (OTA) is a commonly detected mycotoxin in milk, often in conjunction with AFM1 [17, 20, 21]. Zearalenone (ZEN) and its metabolites zearalanone (ZAN), α-zearalanol (α-ZAL), β-zearalanol (β-ZAL), α-zearalenol (α-ZEL), and β-zearalenol (β-ZEL) are also among the frequently reported mycotoxins in milk [17, 20, 22]. Among the trichothecenes family of mycotoxins, deoxynivalenol (DON) and its metabolite de-epoxy-DON [23, 24] as well as T-2 and HT-2 toxins [18] have also been detected in milk. The emerging mycotoxins beauvericin (BEA) and enniatins (ENNs) have recently been detected in milk with high incidence [18, 24, 25].

The presence of multiple types of fungi (mainly *Aspergillus*, *Penicillium*, and *Fusarium* species) in feed makes the co-occurrence of multiple toxins in milk a likely scenario [26]. Mao et al. have reported the presence of different aflatoxins (B1, G1, M1, M2), different zearalenone metabolites (α-ZEL, β-ZEL, α-ZAL, β-ZAL), OTA, and ochratoxin B (OTB) in raw cow milk [17]. In a high-resolution mass spectrometry (HR-MS) screening of bulk milk samples collected from dairy farms using corn silages as the main feed ingredients, a total of 46 mycotoxins from different classes have been identified [24] which demonstrates an ample co-occurrence.

The plant toxins pyrrolizidine alkaloids (PAs) are commonly found in feed, mainly due to the accidental co-harvesting of PA-containing weeds [10, 27, 28]. Several PAs have been detected in the milk of dairy cows [29–31]. Similarly, tropane alkaloids (TAs) such as atropine and scopolamine find their way into milk through contaminated feed [32, 33] and studies have demonstrated the co-occurrence of PAs and TAs in cow milk [30, 31]. In addition, the plant toxins quinolizidine alkaloids (QAs) have been shown to be transferred from feed into milk, as demonstrated by a transfer study [34].

The simultaneous determination of multiple toxins from various chemical classes using multi-method approaches allows for the screening and/or quantification of a wide range of toxins in complex matrices. The sensitive and specific high-performance liquid chromatography coupled to tandem mass spectrometry (HPLC–MS/MS) is considered the state-of-the-art analytical technique for multi-analyte analysis. Examples include the simultaneous determination of pesticides (136 analytes), mycotoxins (23), plant toxins (13), and veterinary drugs (86) in feed, maize, honey, meat, egg, and milk matrices [35]; pesticides (288) and mycotoxins (38) in fruits, cereals, spices, and oil seeds [36]; and > 500 mycotoxins and other secondary metabolites in wheat, maize, figs, dried grapes, walnuts, pistachios, and almonds matrices [37]. In the matrix milk, multi-methods that include varying numbers of mycotoxins (14–46) [17, 24, 38] and plant toxins (53) [30] have been also reported.

The extraction of multiple toxins with diverse physicochemical properties and polarities represents a significant challenge and calls for a “generic” extraction method. The Dilute-and-Shoot and QuEChERS-based (quick, easy, cheap, effective, rugged, and safe) methods are the most widely used “generic” approaches and were thoroughly assessed elsewhere [35, 36, 39]. For the matrix milk, QuEChERS-based methods are the most frequently used for the multi-analyte extraction [18, 21, 40].

In this work, a LC–MS/MS method for the simultaneous determination of a wide range of mycotoxins and plant toxins (110 toxins; 72 mycotoxins and 38 plant toxins) in raw cow milk was developed. The method involved QuEChERS-based extraction and LC–MS/MS analysis using triple quadrupole mass spectrometry. It was validated according to the latest EU regulations for mycotoxins [41] and plant toxins [42] in terms of recovery, precision, and limit of quantification (LOQ). The method has a target LOQ for the regulated AFM1 ≤ 0.025 µg/kg (≤ ½ the ML).

The mycotoxins covered by this method include aflatoxins, trichothecenes (nivalenol and related compounds, T-2 toxin and related compounds, among others), fumonisins, zearalenone and related compounds, beauvericin, enniatins, ochratoxins, *Alternaria* toxins, and ergot alkaloids. The plant toxins covered by this method include tropane alkaloids, pyrrolizidine alkaloids, and quinolizidine alkaloids. To demonstrate its applicability, the validated LC–MS/MS multi-method was applied to 20 milk samples from retail stores and local farms.

## Materials and methods

### Chemicals and standards

Reference standards and isotopically labelled AFM1 (^13^C_17_-AFM1) internal standard (IS) were purchased from the following commercial sources: ASCA GmbH (Berlin, Germany), Biopure, Romer Labs® Inc. (Butzbach, Germany), Biozol GmbH (Eching, Germany), Biomol GmbH (Hamburg, Germany), Cfm Oskar Tropitzsch GmbH (Marktredwitz, Germany), LGC Standards (Wesel, Germany), PhytoLab GmbH (Vestenbergsgreuth, Germany), Phytoplan Diehm & Neuberger GmbH (Heidelberg, Germany), Santa Cruz Biotechnology Inc. (Heidelberg, Germany), Sigma-Aldrich (Schnelldorf, Germany), and Toronto Research Chemicals (TRC, Ontario Canada). The specific details are provided in Table S1. Acetonitrile (ACN), methanol (MeOH), hexane, formic acid (FA), and ammonium formate were of LC–MS grade and purchased from Merck (Darmstadt, Germany). Glycerol was purchased from Sigma-Aldrich (Schnelldorf, Germany) and QuEChERS salts (NaCl, MgSO_4_) were purchased from Agilent Technologies (Waldbronn, Germany). Double-deionized water was obtained using a Milli-Q system from Merck (Merck Millipore, Darmstadt, Germany).

### Milk samples

#### Blank sample for method validation

A bulk tank raw milk sample collected from cows at the BfR experimental farm in Berlin, Germany was used as a blank. Milk samples from the BfR experimental farm and the market were screened for the presence of mycotoxins and plant toxins using LC–MS/MS. It was not possible to find a sample completely free from BEA, ENNs and QAs. The sample with the least amounts of BEA (0.093 µg/kg), ENNA (0.001 µg/kg), ENNA1 (0.006 µg/kg), ENNB (0.057 µg/kg), ENNB1 (0.007 µg/kg), and the QAs angustifoline (0.006 µg/kg), 13a-hydroxylupanine (0.054 µg/kg), isolupanine (0.005 µg/kg), and lupanine (0.050 µg/kg) was selected as a blank and used for method validation. These traces of BEA, ENNs and QAs in blank milk were quantified using the standard addition technique and their levels were considered in any calculations of concentrations when the blank sample was used for generating matrix-matched standard (MMS) solutions and spiking quality control (QC) samples.

#### Milk samples

Overall, 20 milk samples from the BfR experimental farm (raw milk) and local retail stores (whole milk, ≥ 3.5% fat) were analyzed. The samples include 15 conventional and 5 organic samples with the “Bio” label (henceforth will be referred to as organic sample). All samples were stored at − 20 °C until analysis. The samples are listed in Table S2.

### Sample preparation

**Fig. 1:**
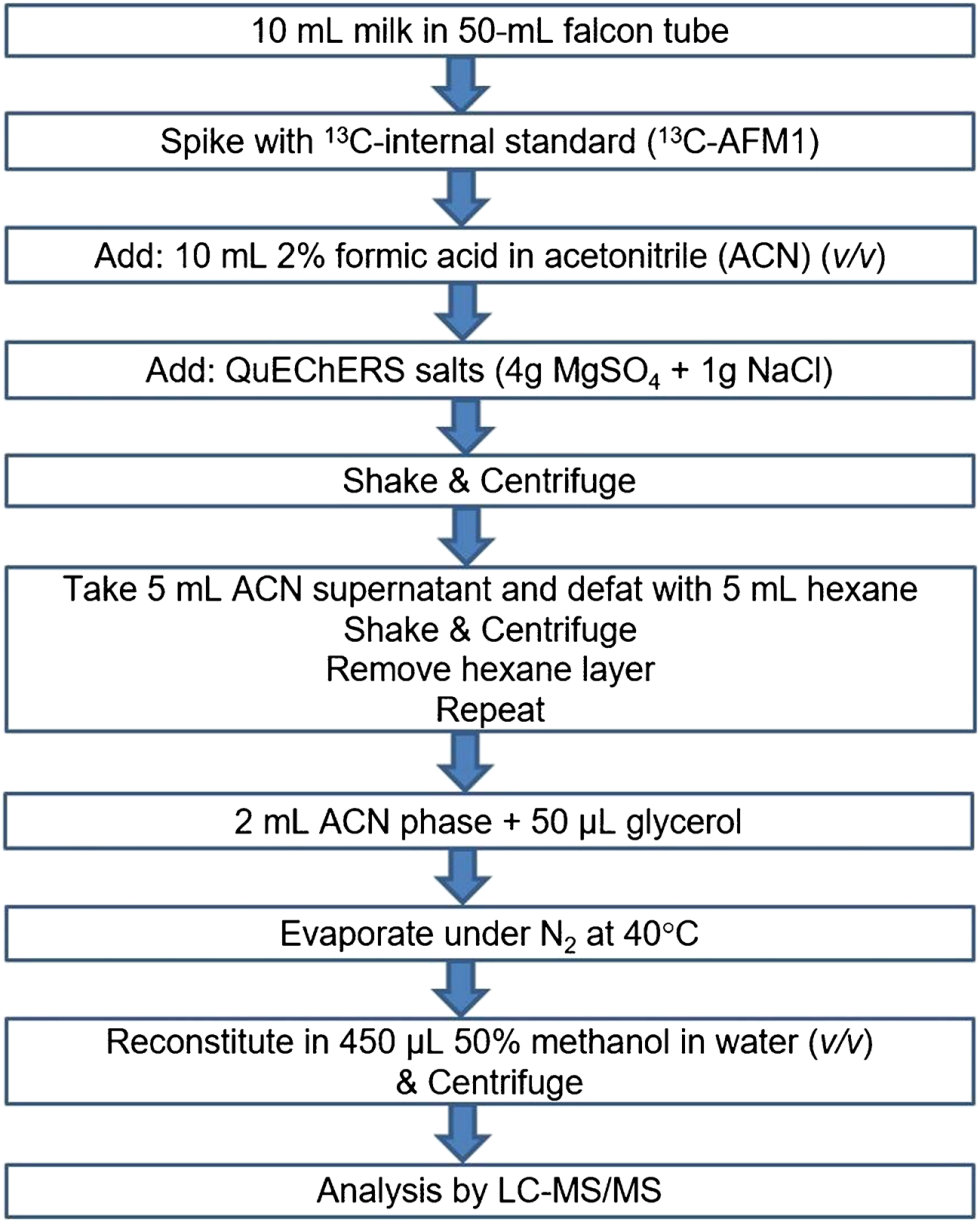
Extraction of mycotoxins and plant toxins from milk

The sample preparation includes QuEChERS-based extraction and is summarized in Fig. 1. Each sample was prepared in duplicate and injected twice. All samples were spiked with ^13^C_17_-AFM1 IS before extraction. Apparatus: Shaker: Multi Reax, Heidolph Instruments (Schwabach, Germany); Centrifuge: Heraeus Megafuge 16, Thermo Fisher Scientific (Waltham, USA); Concentration workstation: TurboVap LV, Caliper Life Sciences (MA, USA). Samples with analyte levels exceeding the upper working range of the method were diluted and reanalyzed.

### Stock and working standard preparation

The multi-analyte stock solution was prepared by combining 19 different solutions: 9 single analyte solutions and 10 mixed solutions containing different numbers of analytes. Due to the variable solubilities of analytes, the solutions were prepared in either ACN, 50% ACN in water (v/v), MeOH, or 50% MeOH in water (v/v). All solutions were stored at − 20 °C. A working standard mixture was freshly prepared by diluting the multi-analyte stock solution with 50% MeOH in water (v/v). The concentrations of the individual standards in the working standard solution are listed in Table S3. For calibration, a series of solutions was prepared in 50% MeOH in water (v/v) and blank extract (matrix-matched calibration) (Table S3).

### LC–MS/MS instrumentation and measurements

Analyses of extracted samples were performed on a Shimadzu Nexera X2 HPLC system, including binary pumps, a degasser, a column oven, an autosampler, and a control unit (Shimadzu Corporation, Duisburg, Germany) coupled to a QTrap 6500 + mass spectrometer (Sciex Germany GmbH, Darmstadt, Germany) equipped with an IonDrive™ Turbo V electrospray ionization (ESI) source. Chromatographic reversed-phase (RP) separation with 5 μL injection volume was achieved on a Waters XBridge Premier BEH C18 column (150 × 2.1 mm, 2.5 μm particle size) with guard column (Waters, Milford, MA, USA) at a flow rate of 0.35 mL/min and a column oven temperature of 40 °C. The binary mobile phase consisted of 5 mM ammonium formate and 0.1% FA in both water (eluent A) and methanol (eluent B). The gradient elution was adopted as follows: 0 min 0% B; 0.75 min 0% B; 1 min 10% B; 2 min 15% B; 9 min 33.5% B; 17 min 77.8% B; 19 min 100% B; 22 min 100% B; 22.1 min 50% B; 23 min 50% B; 23.1 min 0% B; 26 min 0% B. MS detection was conducted using positive and negative electrospray ionization (ESI) modes (polarity switching) and measuring in scheduled multiple reaction monitoring (sMRM) mode using the mass transitions and MS/MS conditions shown in Table S4. The following instrumental settings were applied: curtain gas: 40; collision gas: high; temperature: 300 °C; ion spray voltage: 4500 (− 4000) V; ion source gas 1: 40; ion source gas 2: 40. A diverter valve cut off the flow to the MS ion source before minute 1 and after minute 22.

### Method validation

The method was validated to meet the performance criteria laid down in the EU regulations for mycotoxins [41] and plant toxins [42] analysis. For initial method validation, spiking at two levels (*n* = 4) was conducted. The concentrations of the higher level were twice the lower level. The lower level was then chosen for the ongoing method validation, which was performed in each batch (total *n* = 10). The method validation parameters were determined as follows:

- *Linearity and range:* a series of matrix-matched standard (MMS) solutions in the ranges listed in Table S3 were evaluated.
- *Recovery:* quality control (QC) samples were prepared by spiking blank samples at 2 levels. The concentrations of individual toxins in the spiked samples are shown in Table S3.
- *Precision:* repeatability (RSDr) and within-laboratory reproducibility (inter-day precision, RSD_wR_) were determined for the QC samples at 2 levels.
- *Lowest validated level (LVL):* for official control purposes (checking compliance against maximum, guidance or indicative levels), the EU regulations 2023/2782 and 2023/2783 define the LOQ as the lowest successfully validated level which is “the lowest tested concentration of analyte in a sample material, for which it has been demonstrated that the criteria for recovery, precision, and identification are met”. To avoid confusion with the other definition of LOQ below, this level will henceforth be referred to as the lowest validated level (LVL) in this study. The lowest spiked concentration of the regulated AFM1 was 0.025 µg/kg (= 1/2 ML). The concentrations of individual toxins in the spiked QC samples at LVL are shown in Table S3.
- *Limit of detection (LOD), limit of quantification (LOQ):* for risk assessment and monitoring purposes, it is acceptable to report fit-for-purpose LOQ levels to help generate numerical data for the majority of samples analyzed to avoid left-censored data and hence perform accurate exposure assessment. These fit-for-purpose LOQ levels are generally lower than the LVL used for official control. For this purpose, LOD and LOQ were determined according to the EURL Guidance Document on the Estimation of LOD and LOQ for Measurements in the Field of Contaminants in Feed and Food [43] using spiked blank samples. In short, 10 independent blank samples spiked at the concentration of the estimated LOD were analyzed (Table S3). The spiked concentration of the regulated AFM1 was 0.0025 µg/kg. The standard deviation of signal values of these 10 spiked blanks was used for the estimation of LOD and LOQ. This procedure provides an efficient way to determine the lowest level that can be technically quantified. In contrast, the LVL can be considerably higher than technically feasible, especially if no blank sample is available.
- *Matrix effect:* the response of the MMS solutions was compared to that of standard solutions prepared in 50% MeOH in water (v/v).
- *Specificity and Carry-Over Effects:* several milk samples were tested for the presence of interfering signals with MRM transitions and/or the presence of mycotoxins and plant toxins in non-spiked milk extracts. The chromatographic separation (including testing several columns and LC gradients) and the selection of interference-free MRMs were investigated and optimized during method validation using spiked milk extracts to ensure good chromatographic separation of target analytes from interfering compounds such as isomers or matrix-related ions. HPLC carry-over was estimated by injecting a solvent blank after the highest calibration level. In the analytical batch, 2 solvent blanks were injected after the highest calibration level, and a blank was injected every 10 sample injections. Reagent blank, blank matrix extract, and QC samples were injected at the beginning, middle, and the end of the batch.

### Data analysis

LC–MS/MS data evaluation was performed with MultiQuant Software, ver. 3.0.2 (AB Sciex Germany GmbH, Darmstadt, Germany).

## Results and discussion

### Extraction optimization

In the proposed method, a QuEChERS-based extraction was used, similar to the standard method EN 17641, without immunoaffinity column (IAC) purification. EN 17641 includes extraction of 12 mycotoxins using acidified ACN (1% acetic acid) and water followed by QuEChERS-based extraction. Following phase separation, the ACN supernatant was defatted using hexane and the extract was then evaporated to dryness under nitrogen, reconstituted in MeOH/H_2_O, and analyzed by LC–MS/MS. In the present study, the method was expanded to include more mycotoxins (72 mycotoxins including more trichothecenes, related compounds of ZEN, BEA, ENNs, *Alternaria* toxins and ergot alkaloids) in addition to 38 plant toxins. In the extraction solution, the use of formic acid (FA) was preferred over acetic acid as FA demonstrated more satisfactory recoveries while extracting multiple classes of compounds [35, 36, 39] especially for acidic compounds such as tenuazonic acid (TEA) [44]. Furthermore, the presented method includes the addition of glycerol as a keeper before evaporating the extract. Keepers are high boiling point solvents that are used to minimize the losses of analytes during evaporation [45, 46]. Through preventing complete evaporation of the extract, glycerol prevents the loss of analytes through adsorption and dilutes the remaining FA and thus minimizes the cleavage of labile conjugates such as glucosides and sulfates.

### Method performance and validation

In this study, a LC–MS/MS method was developed and validated for the simultaneous determination of a wide range of mycotoxins and plant toxins in raw cow milk (72 mycotoxins and 38 plant toxins). The validation results were generally in accordance with the criteria specified in the most recent EU guidelines for the analysis of mycotoxins and plant toxins (Tables S5 and S6 for validation at level 1 (LVL, ongoing validation, *n* = 10) and level 2 (2 × LVL, initial validation, *n* = 4), respectively). From this point forward in the manuscript, the validation data of the ongoing validation will be used.

#### Identification

The retention time of the analyte in the extract should match that of the matrix-matched calibration standard with a tolerance of ± 0.1 min. Peaks of both MRM transitions in the extracted ion chromatograms must fully overlap. The ion ratio of MRM transitions from sample extracts should be within ± 30% of the average of ion ratios of the calibration standards from the same sequence. Hereafter, a sample is considered positive for a certain analyte when the identification criteria are met in all four injections of the prepared sample.

#### Linearity and range

The analytes showed good linearity and all calibration levels have fulfilled the criterion of within ± 20% deviation from the true concentrations using back-calculation. The calibration linearity range and the equivalent working concentration range in milk (µg analyte/kg milk) for individual analytes are shown in Table S7.

#### Recovery

**Fig. 2:**
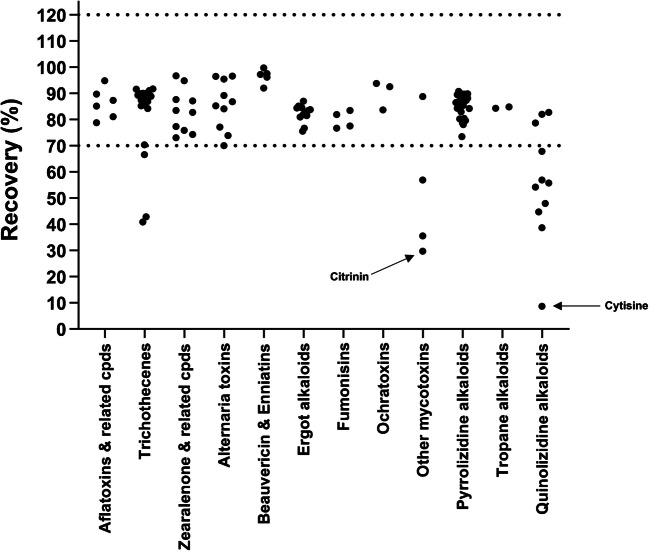
Summary of average recoveries of extractions obtained under within-laboratory reproducibility conditions (*n* = 10)

The method showed excellent recoveries for the majority of analytes (Fig. 2). The average recoveries of individual analytes are shown in Table S5. Figure 2 shows that 96 out of 110 (87%) analytes have average recoveries within the target range (70–120%). The average recoveries of 6 analytes are within 50–70%, another 6 analytes within 30–50%, and only 2 analytes < 30%. For analytes with average recoveries < 50%, the proposed method can only be used for screening. In the case of positive results in milk samples, these analytes can then be quantified using analyte-specific methods. From the mycotoxins, only 6 analytes (nivalenol (NIV), roquefortine C, DON-3-glucoside (DON-3G), verrucarol, phomopsin A, and citrinin (CIT)) have recoveries < 70%, with only CIT having recovery < 30%. The method development has demonstrated that the losses of analytes during extraction mainly occur during phase separation (QuEChERS) and/or evaporation steps. For NIV, this relatively polar analyte is mainly lost during QuEChERS-induced phase separation, as previously reported [47]. Without phase separation, the recovery of NIV was acceptable (≈ 85%) (data not shown). Similarly, DON-3G was shown to be lost to the aqueous phase during phase separation. Simple extraction (without QuEChERS-induced phase separation) showed excellent recovery (≈ 100%) for DON-3G (data not shown). Acid-induced cleavage does not play a significant role in the losses of DON-3G. This can be demonstrated by the acceptable recoveries for the glucoside and sulfate conjugates of alternariol (AOH), alternariol monomethyl ether (AME), and ZEN. The polarity of DON-3G is considerably higher than that of the glucoside and sulfate conjugates of AOH, AME, ZEN, as reflected by their respective chromatographic retention times (Table S4). The losses of roquefortine C, verrucarol, and CIT were shown to occur during the evaporation step. Acceptable recoveries (90%, 87%, and 86% for roquefortine C, verrucarol, and CIT, respectively) were obtained when the QuEChERS extract was directly diluted and analyzed without evaporation (data not shown). On the other hand, phomopsin A was partially lost both during phase separation and evaporation.

From the plant toxins, all the PAs and TAs have recoveries within the target range (70–120%). However, the recoveries of QAs showed considerable variations, where only 3 QAs (angustifoline, isolupanine and lupanine) have acceptable recoveries (70–120%), 4 QAs within 50–70%, 3 QAs within 30–50%, and only one (cytisine) < 30%. The highly polar QAs were probably lost during QuEChERS-induced phase separation or during evaporation. Simple extraction of milk (without QuEChERS) showed acceptable recoveries (70–120%) for all QAs [34].

#### Precision

**Fig. 3:**
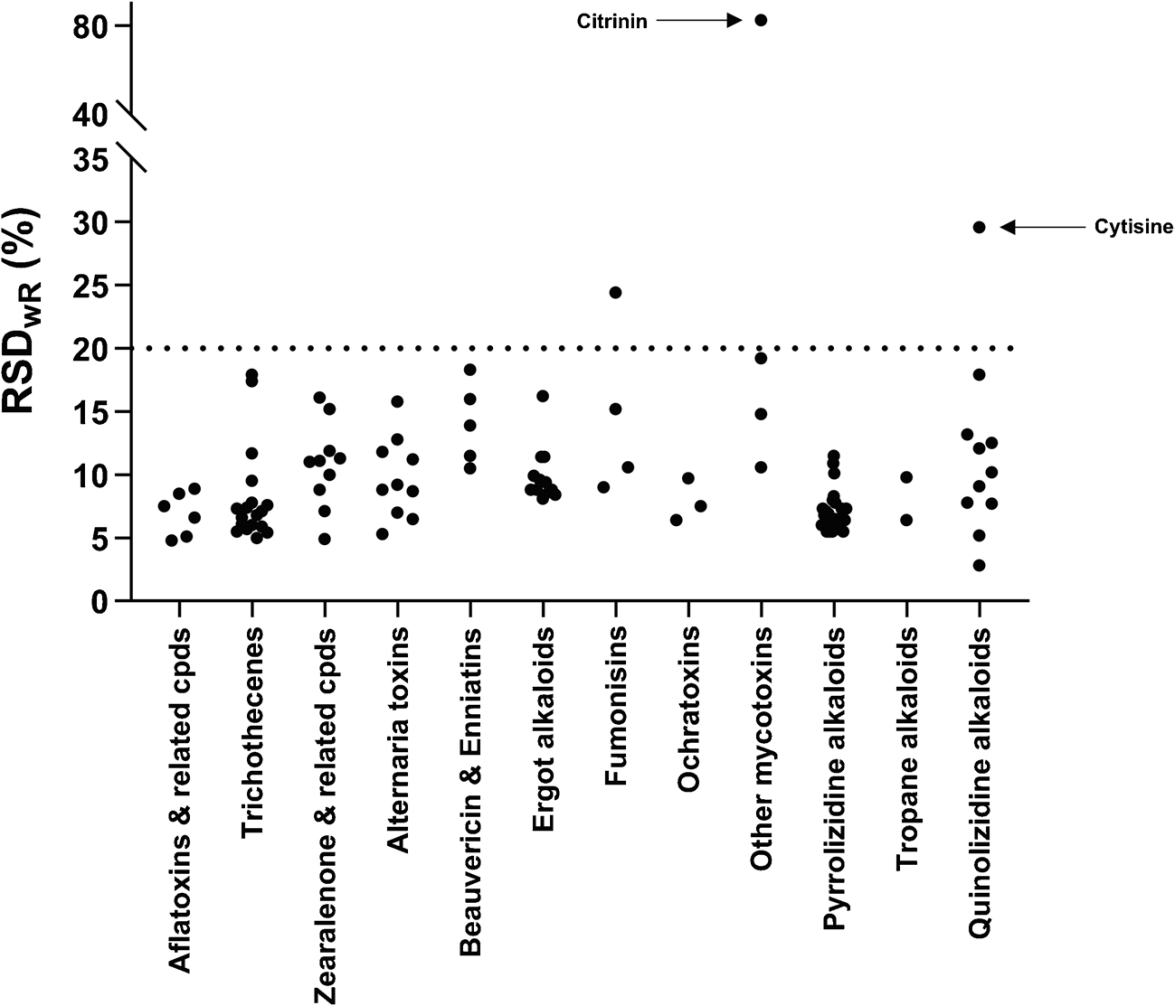
Summary of within-laboratory reproducibility (intermediate precision) (RSD_wR_) (*n* = 10)

The method demonstrated excellent precision with repeatability (RSDr) meeting the target criterion (≤ 20%) for all analytes (Figure S1) and 97% (107 out of 110) of the analytes having within-laboratory reproducibility (RSD_wR_) within the target range (≤ 20%) (Fig. 3). The RSDr and RSD_wR_ of individual analytes are shown in Table S5. Only one mycotoxin, hydrolyzed fumonisin B1, has RSD_wR_ within the range 20–25%. The mycotoxin CIT and plant toxin cytisine are the only analytes with RSD_wR_ > 25%. CIT and cytisine show also the lowest recoveries among mycotoxins and plant toxins, respectively, which indicate the inconsistent extraction of these analytes using the present method.

#### Matrix effect

The suppression/enhancement of analyte ionization (signal) due to the co-eluting matrix components has been studied. The data for individual analytes in Table S5 show that 78 out of 110 analytes have signal suppression/enhancement within the range 100 ± 20%. Only 5 analytes show elevated signal enhancement (> 120%) whereas 27 analytes show elevated signal suppression (< 80%). To compensate for the matrix effects, matrix-matched calibration was implemented in the proposed method. MMS eliminates the need for matrix effect correction as the matrix effects will be covered by the recovery criterion.

#### Sensitivity

The LOD, LOQ, and LVL data for individual analytes are shown in Table S5. To meet the ML for the regulated aflatoxin M1 in milk (0.05 µg/kg), the method had a target LVL for AFM1 ≤ 0.025 µg/kg (≤ ½ the ML). Experimentally, the lowest validated level (LVL) for AFM1 was 0.025 µg/kg, whereas the LOQ was 0.0035 µg/kg. This makes the proposed multi-method suitable for the confident determination of the regulated AFM1 in milk samples.

#### Specificity and carry-over effects

Quality assurance included the check for false positives. Despite testing several samples for interfering signals, it was not possible to find a sample completely free from BEA, ENNs, and QAs, and the sample with the least amounts of BEA, ENNs, and QAs was selected as a blank and used for method validation. The combination of the selected column, the careful selection of MRMs (also the selection of adducts), a relatively long gradient, and the selection of the suitable polarity resulted in a good chromatographic separation of target analytes from interfering compounds such as isomers (e.g., ergot alkaloids (EAs), PAs, QAs, fumonisin B2 and B3…etc.) and/or matrix-related ions. From EAs, only ergotamine and ergotaminine were not chromatographically separated and can therefore only be determined as a sum. The rest of EAs were well separated. The isobaric PAs in the method were also well separated (e.g., echimidine-N and heliosupine-N have RT of 10.88 and 12.20 min, respectively). HPLC carry-over between consecutive HPLC runs and the presence of analyte signals in reagent and matrix blanks and in the presence of other analytes were investigated in every batch. No interference above LOD was found for any of the analytes.

#### Overall performance of the multi-method

The presented LC–MS/MS multi-method enables the simultaneous determination and quantification of mycotoxins and plant toxins in milk, significantly enhancing sample throughput. Using a single extraction protocol and a single LC–MS/MS run for all target analytes helps minimize labor, reagent consumption, instrumentation time, and operational costs, thereby reducing overall analysis costs. Its application in monitoring analyses would therefore minimize time, effort, and cost and maximize the information output per sample. Despite the inherent complexity of the milk matrix and the diverse physicochemical properties and polarities of mycotoxins and plant toxins, the presented method could still fulfill the requirements of the latest EU regulations for analytical methods for mycotoxins and plant toxins analysis for the majority of analytes. The multi-method delivered excellent recoveries (87% of the analytes within the target 70–120%), precision (97% of the analytes have RSD_wR_ ≤ 20%), and LOQ for the regulated AFM1 (0.0035 µg/kg) that is sevenfold lower than the target LOQ of ≤ 0.025 µg/kg (≤ ½ the ML). In comparison to the published multi-methods for the determination of mycotoxins or plant toxins in the milk matrix, the proposed multi-method shows comparable (sometimes better) validation data (despite covering a larger number of analytes). As an example, for the regulated AFM1, the proposed multi-method has a recovery, RSD_wR_, and LOQ of 95%, 7.5%, and 0.0035 µg/kg, respectively. Other QuEChERS-based multi-methods showed 94%, 7.0%, and 0.01 µg/kg [40] and 108%, 7.2%, and 0.01 µg/kg [38]. The detailed comparison is listed in Table S8. These robust validation results demonstrate the capabilities of the multi-method in overcoming matrix challenges, making it a reliable tool for comprehensive mycotoxins and plant toxins monitoring and risk assessment, even in challenging matrices. Compared to the methods in the literature, to the best of our knowledge, this is the first validated method for the milk matrix that includes > 100 mycotoxins and plant toxins. The presented method covers the largest number of mycotoxins (72) and, for the first time, includes the three groups of plant toxins: tropane, pyrrolizidine, and quinolizidine alkaloids.

### Application to milk samples

**Fig. 4:**
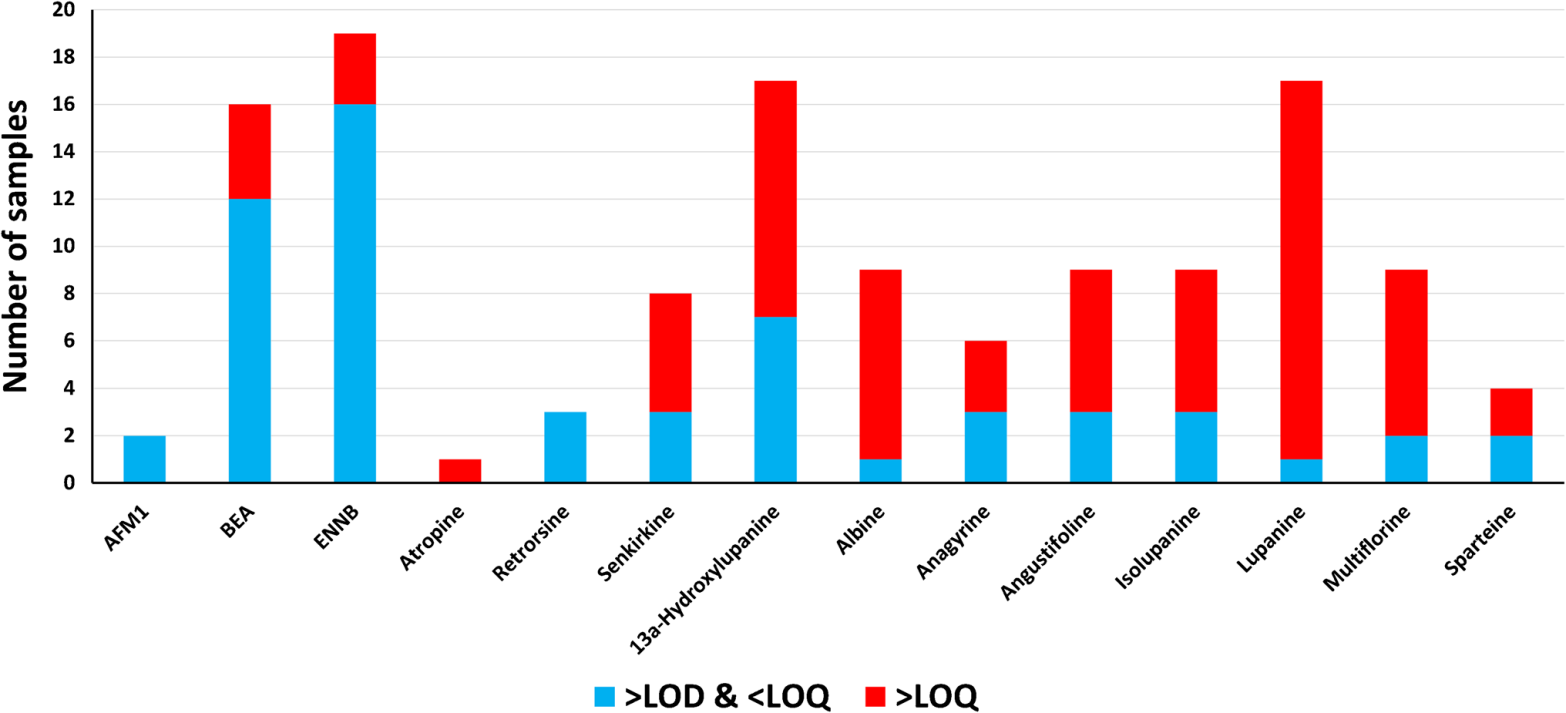
Occurrence of mycotoxins and plant toxins in the analyzed milk samples from Germany (*n* = 20)

The summary of positive results (results above LOD) in milk samples is shown in Fig. 4, while Table 1 compares positive results between conventional and organic milk samples. The results of individual analytes in milk samples are detailed in Table S9.

#### Occurrence of mycotoxins

##### Aflatoxins

AFM1 was detected in 2 (10%) samples and both occurrences were below LOQ. All samples were compliant with EU legislation (all < ML of AFM1 = 0.05 µg/kg).

##### Emerging mycotoxins

Beauvericin (BEA) was detected in 16 (80%) samples. Four conventional milk samples contained levels above the LOQ of BEA, with the highest concentration of 0.293 µg/kg (range: 0.230–0.293 µg/kg). The levels of BEA in our study (samples from Germany) are in agreement with previous reports from Poland (range: 0.101–1.93 µg/kg) [25] and Portugal (0.090–0.810 µg/kg) [18]. Enniatin B (ENNB) was also detected in 19 (95%) samples. The highest concentrations of ENNB in conventional and organic milk samples were 0.128 and 0.154 µg/kg, respectively (Table 1). These results are in agreement with the study from Poland (0.157–0.587 µg/kg) [25]. However, the study from Portugal reported relatively higher levels of ENNB (4.36–10.6 µg/kg) than in our study and also the presence of quantifiable amounts of enniatin A (9.78–16.3 µg/kg) [18]. It has to be noted that traces of enniatin B1 (20 samples, 100%), enniatin A (*n* = 8, 40%), and enniatin A1 (*n* = 12, 60%) were also identified at levels below the LOD in both conventional and organic milk samples (data not shown) (data below LOD are only indicative. The identification criteria (RT and ion ratio) were met in all duplicate injections, thus ensuring the presence of the analyte. For practical reasons related to the complexity of the multi-analyte standard solution, the spiking level used to determine the LOD in this study was set higher than the actual limit of detection resulting in slightly higher LOD values than technically feasible). Due to data gaps in the toxicological and toxicokinetic data, the tolerable daily intake (TDI), acute reference dose (ARfD), and/or maximum levels for BEA and ENNs in humans and animals have not yet been established. Therefore, a full risk assessment is not available at the moment [48, 49]. However, the levels of BEA and ENNs in the milk samples in our study were orders of magnitude lower than levels found in cereals (levels of up to 6402 and 18,300 µg/kg for BEA and ENNB, respectively) and are therefore unlikely to indicate concern for human health [48].

#### Occurrence of plant toxins

##### Quinolizidine alkaloids

**Fig. 5:**
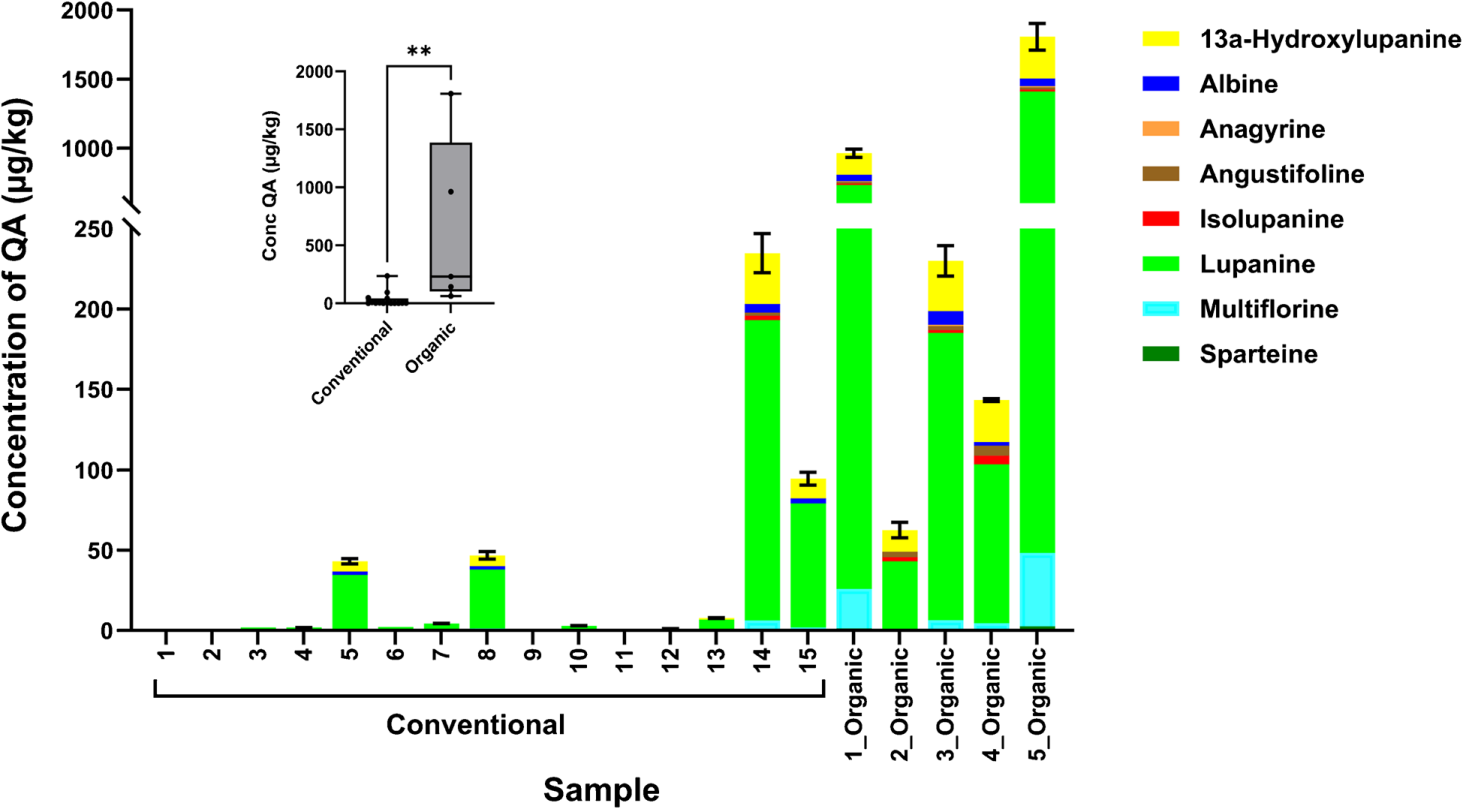
The contents of quinolizidine alkaloids in conventional (*n* = 15) and organic (*n* = 5) milk samples. The inset shows the comparison of the sum of quinolizidine alkaloids in the conventional and organic groups (**p < 0.005)

The quinolizidine alkaloids (QAs) 13a-hydroxylupanine and lupanine were detected in 17 (85%) milk samples. Albine, angustifoline, isolupanine, and multiflorine were detected in 9 (45%) samples, while anagyrine and sparteine were detected in 6 (30%) and 4 (20%) samples, respectively. Lupanine was, by far, the most abundant among all QAs (the highest maximum, median and mean concentrations) as shown in Table 1 and Fig. 5. The percentages of positive samples (above LOD) were generally higher within the organic milk group. Furthermore, the percentages of samples above LOQ were also higher within the organic milk group. All organic samples contained 13a-hydroxylupanine, albine, angustifoline, isolupanine, lupanine, and multiflorine, with above LOQ levels of 13a-hydroxylupanine, angustifoline, isolupanine, and lupanine in all samples. The levels of QAs were also significantly higher (*p* < 0.005) in organic samples compared to conventional samples (Table 1 and Fig. 5). The highest concentrations of lupanine in conventional and organic milk samples were 187 (range: 1.01–187) and 1361 (range: 43.0–1361) µg/kg, respectively. Similar differences between conventional and organic samples are also shown for 13a-hydroxylupanine, albine, angustifoline, isolupanine, and multiflorine (Table 1 and Fig. 5) which all indicate that organic milk samples show higher contamination levels of QAs than conventional samples.

EFSA and the German Federal Institute for Risk Assessment (BfR) provided a preliminary assessment of the risks associated with QAs in feed and food, particularly focusing on lupins and lupin-derived products [50, 51]. Engel et al. conducted a transfer study and were able to demonstrate the transfer of (QAs) to milk. In addition, their toxicological assessment (applying margin of exposure (MoE) approach using the dose of 0.16 mg sparteine/kg BW as a reference point) indicated a potential health concern when milk with levels of QAs found in their study was consumed [34]. In the present study, two organic milk samples (samples “1_Organic” and “5_Organic”) contain levels of lupanine (708 and 1361 µg/kg, respectively) comparable to the average levels reported in the transfer study conducted by Engel et al. (range in milk: 388–1738 µg/kg) (using blue sweet lupin seeds in the feed, the cows were fed a total QAs of 1774 and 3549 mg/day). The presence of QAs in milk from the German market was later confirmed in a report on food safety monitoring issued by the German Federal Office of Consumer Protection and Food Safety (BVL). In agreement with our findings, the maximum levels of lupanine in the BVL monitoring report were 35.5 and 1029 µg/kg in conventional and organic samples, respectively. However, the BVL concluded that elevated QA levels were observed only in individual samples, suggesting that retail cow’s milk is not a major source of human QA exposure and that further investigations are recommended [52]. Considering that this study as well as the aforementioned studies showed that more than 50% of the total QA levels could be attributed to lupanine, lupanine may be considered a marker substance for QAs in milk.

##### Pyrrolizidine and tropane alkaloids

Senkirkine was the only pyrrolizidine alkaloid detected at levels above LOQ in 5 (25%) samples. The highest concentrations of senkirkine in conventional and organic milk samples were 0.248 (range: 0.043–0.248) and 0.270 µg/kg, respectively (Table 1). Traces of the pyrrolizidine alkaloids retrorsine (*n* = 3, 15%, < 0.021 µg/kg) and senkirkine (*n* = 8, 40%) were detected in both milk types. Similar results were previously reported. In the study of Mulder et al., the range of detected PAs was 0.050–0.170 µg/kg with senkirkine showing the highest level [53]. Traces of retrorsine (< 0.039 µg/kg) and senkirkine (< 0.034 µg/kg) were also reported in another study [30]. The tropane alkaloid atropine was detected in a single conventional milk sample with a concentration of 0.037 µg/kg. In a recent study, atropine was also detected in milk samples from Germany (range: 0.028–0.066 µg/kg) [31]. However, levels of PAs and TAs in milk samples are generally low in comparison to the ML in herbal infusions (liquid products) (1.0 and 0.2 µg/kg for sum of PAs and sum of TAs, respectively) [13].

**Table 1:** Occurrence of mycotoxins and plant toxins in 20 analyzed milk samples from Germany

	Conventional milk (*n* = 15)							Organic milk (*n* = 5)						
Analyte	Rate positive samples (> LOD) (%)	# samples > LOD and < LOQ	# samples > LOQ	Max (µg/kg)	Range (µg/kg)	Median (µg/kg)	Mean (µg/kg)	Rate positive samples (> LOD) (%)	# samples > LOD and < LOQ	# samples > LOQ	Max (µg/kg)	Range (µg/kg)	Median (µg/kg)	Mean (µg/kg)
AFM1	13	2	0	-	-	-	-	0	0	0	-	-	-	-
BEA*	93	10	4	0.293	0.230–0.293	0.248	0.255	40	2	0	-	-	-	-
ENNB*	93	13	1	0.128	-	-	-	100	3	2	0.154	0.135–0.154	0.145	0.145
Atropine*	7	0	1	0.037	-	-	-	0	0	0	-	-	-	-
Retrorsine^§^	13	2	0	-	-	-	-	20	1	0	-	-	-	-
Senkirkine*	40	2	4	0.248	0.043–0.248	0.149	0.147	40	1	1	0.270	-	-	-
13a-Hydroxylupanine*	80	7	5	31.6	1.04–31.6	6.79	11.6	100	0	5	303	13.6–303	31.2	106
Albine*	27	0	4	5.37	2.04–5.37	2.59	3.15	100	1	4	55.7	2.21–55.7	27.6	28.3
Anagyrine*	13	2	0	-	-	-	-	80	1	3	7.71	0.929–7.71	3.95	4.20
Angustifoline*	27	3	1	1.86	-	-	-	100	0	5	16.6	2.44–16.6	6.17	8.23
Isolupanine*	27	3	1	2.90	-	-	-	100	0	5	14.3	1.82–14.3	5.39	6.95
Lupanine*	80	1	11	187	1.01–187	4.29	32.4	100	0	5	1361	43.0–1361	179	478
Multiflorine*	27	1	3	6.21	1.25–6.21	1.79	3.09	100	1	4	45.9	4.54–45.9	15.5	20.3
Sparteine*	7	1	0	-	-	-	-	60	1	2	2.49	1.25–2.49	1.87	1.87

*Concentrations are corrected for recovery

^§^May contain the possible co-eluting isomer usaramine. Usaramine was not included in the validated method

#### Co-occurrence of Toxins

The findings showed the co-occurrence of multiple toxins in milk samples with at least 2 analytes occurring together per sample and a maximum of 12 analytes (Figure S2). The emerging mycotoxins BEA and ENNB and the QAs 13a-hydroxylupanine and lupanine are present in the majority of samples. Generally, the emerging mycotoxins (BEA and ENNs) and QAs are the most co-occurring toxins.

## Conclusions

The aim of this work was to develop and validate a multi-method for the simultaneous quantitative determination of 72 mycotoxins and 38 plant toxins in cow milk and to investigate the occurrence of these toxins in cow milk samples. The presented LC–MS/MS multi-method was validated according to the EU regulations 2023/2782 and 2023/2783 and demonstrated excellent validation results in terms of recovery (87% of the analytes within the target 70–120%) and precision (97% of the analytes have RSD_wR_ ≤ 20%). The method also demonstrated sufficient sensitivity and the obtained LVL and LOQ were below the maximum level for the regulated AFM1. These validation results highlight the capabilities of the method to overcome the complexity of the milk matrix and the variable physicochemical properties of the analytes. This is the first validated multi-method for the milk matrix that includes such a number of mycotoxins and plant toxins. The application of the validated method to milk samples showed the frequent occurrence of emerging mycotoxins (especially BEA and ENNB) and quinolizidine alkaloids (especially lupanine and 13a-hydroxylupanine). The pyrrolizidine alkaloid senkirkine was detected in almost half of the samples. QAs (especially lupanine) were detected at much higher concentration levels than other toxin groups. Apart from QAs, only trace levels of mycotoxins and plant toxins were found. However, the potential health effects associated with chronic exposure to such low levels of these toxins and their potential combined effects remain uncertain and require further research. The proposed method proved to be a reliable and efficient tool for the simultaneous determination of multiple mycotoxins and plant toxins in milk and could be used for routine monitoring to enhance productivity while saving time, effort, and cost.

## Supplementary Information

Below is the link to the electronic supplementary material.

Supplementary file1 (DOCX 164 KB)

Supplementary file2 (XLSX 107 KB)

## Data Availability

The data supporting the conclusions of this article will be made available from the authors by request.
