# Supplementary material for: A validated LC–MS/MS multi-method for the determination of 110 mycotoxins and plant toxins in cow milk and application to samples from Germany
Source: Anal Bioanal Chem. 2025 Aug 7;417(22):5047–59. doi: 10.1007/s00216-025-06024-6 (PMC12401758; doi:10.1007/s00216-025-06024-6)
Supplement: Supplementary file 1 — Supplementary file1 (DOCX 164 KB) [file 216_2025_6024_MOESM1_ESM.docx]

**A validated LC-MS/MS multi-method for the determination of 110 mycotoxins and plant toxins in cow milk and application to samples from Germany**

Ahmed H. El-Khatib*, Arnold Bahlmann, Christoph Hutzler, Stefan Weigel

Reference Centre for Food and Feed Analysis, German Federal Institute for Risk Assessment (BfR), Max‑Dohrn‑Str. 8‑10, 10589 Berlin, Germany

* Correspondence: ahmed.el-khatib@bfr.bund.de (A.H.E.-K.)


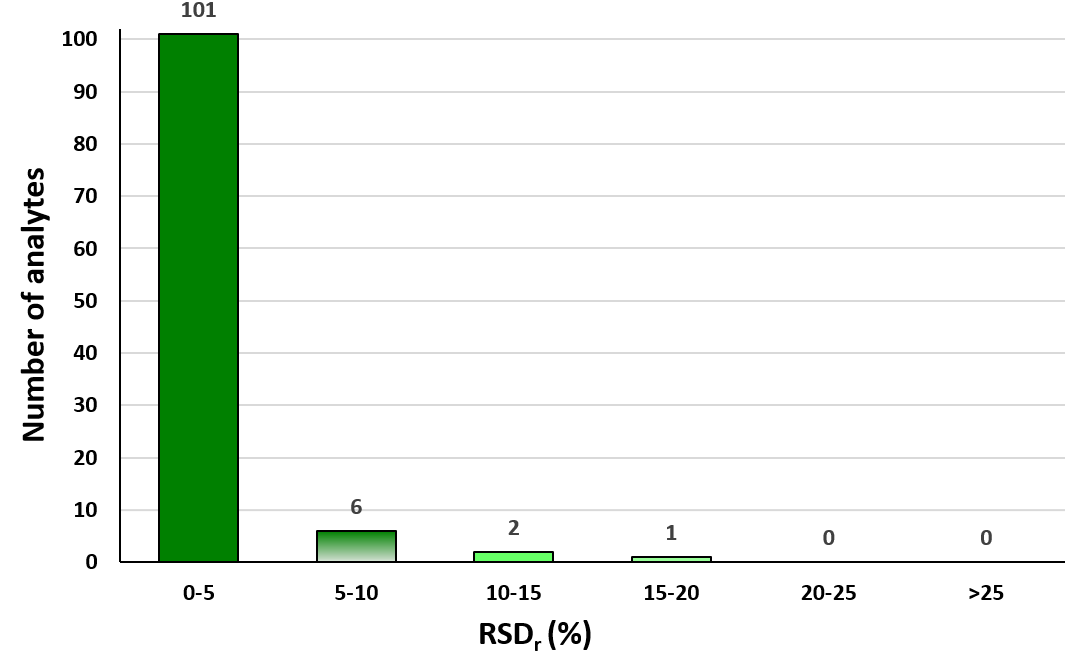


**Figure S1**. Summary of repeatability (RSD_r_) (n=6).


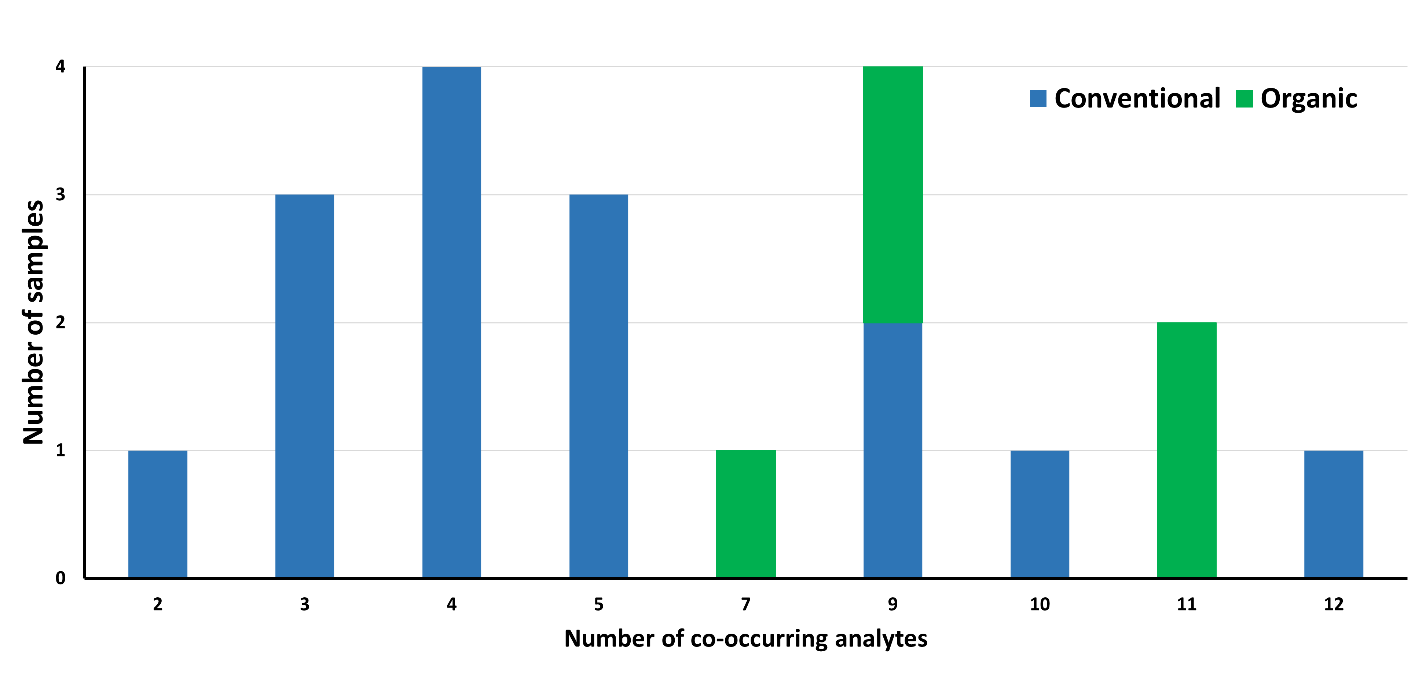


**Figure S2**. Co-occurrence of toxins in conventional (n=15) and organic (n=5) milk samples.
